# Genomic imbalances pinpoint potential oncogenes and tumor suppressors in Wilms tumors

**DOI:** 10.1186/s13039-016-0227-y

**Published:** 2016-02-24

**Authors:** A. C. V. Krepischi, M. Maschietto, E. N. Ferreira, A. G. Silva, S. S. Costa, I. W. da Cunha, B. D. F. Barros, P. E. Grundy, C. Rosenberg, D. M. Carraro

**Affiliations:** International Research Center, AC Camargo Cancer Center, São Paulo, Brazil; Institute of Biosciences, University of São Paulo, São Paulo, Brazil; Brazilian Biosciences National Laboratory, National Center for Research in Energy and Materials, Campinas, São Paulo, Brazil; Department of Surgical and Investigative Pathology, AC Camargo Cancer Center, São Paulo, Brazil; Alberta Health Services, Cancer Control Alberta, Alberta, Canada

**Keywords:** Wilms tumor, Array-CGH, Copy number alteration, CNA, Relapse, 1q21.1-q23.2 gain

## Abstract

**Background:**

Wilms tumor (WT) has a not completely elucidated pathogenesis. DNA copy number alterations (CNAs) are common in cancer, and often define key pathogenic events. The aim of this work was to investigate CNAs in order to disclose new candidate genes for Wilms tumorigenesis.

**Results:**

Array-CGH of 50 primary WTs without pre-chemotherapy revealed a few recurrent CNAs not previously reported, such as 7q and 20q gains, and 7p loss. Genomic amplifications were exclusively detected in 3 cases of WTs that later relapsed, which also exhibited an increased frequency of gains affecting a 16.2 Mb 1q21.1-q23.2 region, losses at 11p, 11q distal, and 16q, and *WT1* deletions. Conversely, aneuploidies of chromosomes 13 and 19 were found only in WTs without further relapse. The 1q21.1-q23.2 gain associated with WT relapse harbours genes such as *CHD1L*, *CRABP2*, *GJA8*, *MEX3A* and *MLLT11* that were found to be over-expressed in WTs. In addition, down-regulation of genes encompassed by focal deletions highlighted new potential tumor suppressors such as *CNKSR1*, *MAN1C*1, *PAQR7* (1p36), *TWIST1*, *SOSTDC1* (7p14.1-p12.2), *BBOX* and *FIBIN* (11p13), and *PLCG2* (16q).

**Conclusion:**

This study confirmed the presence of CNAs previously related to WT and characterized new CNAs found only in few cases. The later were found in higher frequency in relapsed cases, suggesting that they could be associated with WT progression.

**Electronic supplementary material:**

The online version of this article (doi:10.1186/s13039-016-0227-y) contains supplementary material, which is available to authorized users.

## Background

Wilms tumor (WT) is the most common type of malignant renal cancer in childhood, with an incidence of 7.7 in 1 million children between 0 and 14 years of age in Western populations [1, 2]. WTs exhibit a triphasic histology that recapitulates the fetal kidney development [3, 4], and similarly, gene expression profiling of isolated components mimics the ongoing process of nephrogenesis [5].

Earlier cytogenetic studies of patients with WAGR (MIM#194072), a syndrome characterized among others by susceptibility to Wilms tumor, revealed constitutional deletions on chromosome 11p13 affecting several contiguous genes (reviewed in [6]) including *WT1* [7, 8]. Latter on, cytogenetic and molecular studies of tumor material from WT sporadic cases showed the presence of somatic inactivating mutations or deletions of *WT1* in up to 15 % of the cases [9–11].

Other somatic alterations have been causally related to WT such as activating mutations in *CTNNB1* [10, 12], mutation/deletion of *WTX* [11, 13], and loss of imprinting of *IGF2* [9, 11, 14]. Recent molecular cytogenetic studies of tumoral samples have identified other genomic regions harboring genes supposedly associated with WT development, as exemplified by *HACE1* disruption at 6q21 [15], and a 2q37 deletion encompassing the *miR*-*562* [16]. Somatic deletions of *DIS3L2* have also been identified in a group of WT, and germline mutations of this gene results in Perlman syndrome that also presents increased WT susceptibility [17]. More recently, recurrent somatic mutations in *DROSHA* (p.E1147K) as well as in other genes from the microRNA biogenesis machinery (*DGCR8*, *DICER1*, *XPO5* and *TARBP2*) were found in up to 12 % of WTs [18–21]. Additionally, somatic mutations in *SIX1/SIX2* were found in a subgroup of WT presenting high proliferative potential [19]. Because *SIX1* and *DROSHA* mutations were found to be heterogeneous events within primary tumors, both spatially and temporally, it was speculated if their co-occurrence were positively associated with tumor progression rather than tumor onset [22].

Somatic loss of heterozygosity (LOH) at 1p, 11q, 16q, and 22q, and deletions at 12q and 18q were correlated with an adverse outcome [23–26]. In clinical practice, combined LOH of 1p and 16q are used as markers of poor outcome for chemotherapy-naive tumors [23, 24]; however, they are detected in a very small subset of WT patients. In addition to the description of 1p, 1q, 3p, 3q, and 14q imbalances occurring at higher frequency in relapsing tumors than in other tumors [27], copy number gains at 1q have also been associated with poor prognosis in patients with favorable WT histology [28, 29]. A study reported that 1q gain has limited prognostic value for risk stratification in pre-treated WT [30]; however, it has been questioned whether the sample size was large enough and if the parameters used for defining 1q gain were validated to draw this conclusion [31].

This study was designed to assess the genomic copy number alterations profile (CNA) of WTs, aiming to identify genetic markers associated with WT, in particular those with clinical and prognostic importance.

## Results

### Characterization of copy number alterations in Wilms tumors

Array-CGH analysis detected a total of 350 CNAs in all 50 WT samples (mean of 7 CNAs per tumor genome), ranging from focal rearrangements (70 kb–5 Mb) to chromosome-arm alterations, and whole-chromosome aneuploidies. Full and summarized descriptions of the array-CGH data can be found in Additional file 1: Tables S1 and S2. We performed statistical analyses comparing WTs with and without relapse regarding the number, distribution and type (gain, high-copy gain, loss, homozygous loss) of CNAs. Genomic losses were more frequent in the relapse group (*p* = 0.016, Fisher exact test) than in the group without relapse, whereas high gains (>5 copies) were detected exclusively in the group of tumors from patients who relapsed (3 cases).

**Fig. 1:**
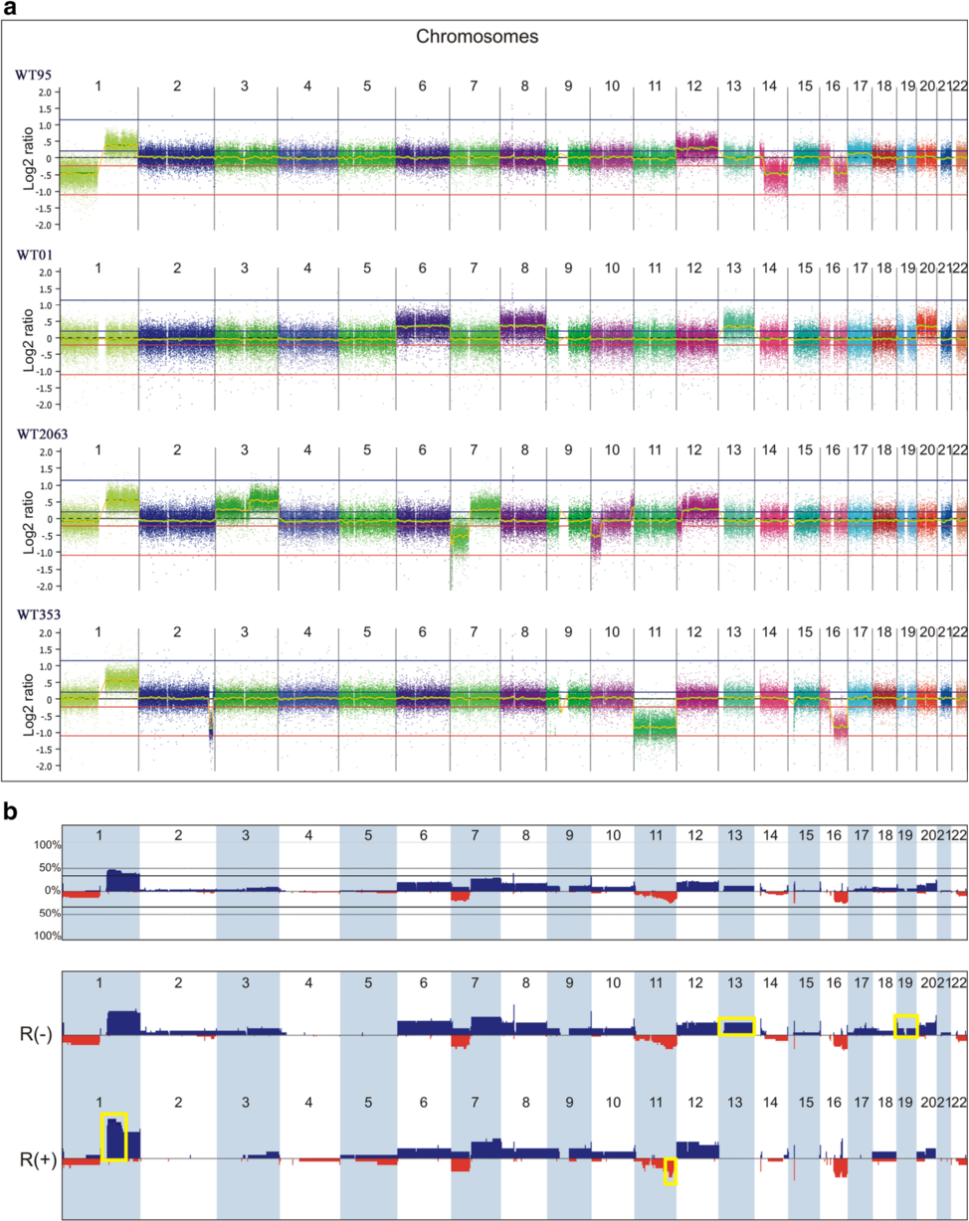
Copy number profiles of sporadic Wilms tumors. All chromosomes are displayed from the short to the long arms. Images adapted from Nexus Copy Number software, Biodiscovery. **a** Array-CGH profile of four selected Wilms tumor samples. **b** Global profile of copy number alterations (CNA) and respective frequencies in the 50 WT samples (*upper panel*) and the CNA distribution according to the occurrence of relapse (*bottom panels*). Genomic gains are indicated by *blue bars*, losses by *red bars* and *yellow boxes* mark the alterations detected in higher frequency or exclusively in each group; relapsed patients (R+), non-relapsed patients (R-)

Typically, WTs present few alterations indicating low chromosomal instability. The log_2_ ratios for most alterations were in a range consistent with heterozygous losses or gains (>0.5 or <−0.5), suggestive of a low level of intra-tumor heterogeneity. As an example, Fig. 1a shows the array-CGH results of four Wilms tumors; each lane shows the copy number profile of all chromosomes for one sample. Figure 1b summarizes the copy number findings detected in the WT cohort and their respective frequencies, showing the full cohort (upper panel) as well as tumors grouped according to the occurrence of relapse (bottom panel). Gains affecting 1q were observed in >50 % of the tumors, but frequent CNAs (>15 % of the WT group) included gains of 7q and 20q, and losses at 1p, 7p, 11q, and 16q, in addition to whole-chromosome aneuploidies of 6, 8, 12, and 20 (gains), and 22 (losses).

WTs derived from patients who later relapsed carried few genomic alterations detected in higher frequency than the group without relapse, such as 1q gain (with a peak at 1q proximal) and losses at 11p (with a peak encompassing the *WT1* gene), 11q distal, and 16q (Fig. 1b, bottom panels). Conversely, aneuploidies of chromosomes 13 and 19 (gains) were exclusively detected in the WT group without relapse.

### Recurrent chromosomal alterations e minimum common regions

Table 1 describes six recurrent (frequency >15 % in the entire WT group) chromosomal rearrangements and their frequencies according to relapse status.

**Table 1:** Six recurrent chromosomal rearrangements (frequency >15 % in the entire WT group) and microdeletions of *WT1* and *WTX* genes according to the relapse status

WT group	Recurrent rearrangements (chromosome arm/focal)						Whole-chromosome aneuploidies					Microdeletion of known WT genes	
	1p(−)	1q(+)	7p(−)	7q(+)	11q(−)	16q(−)	chr6	chr8	chr12	chr20(+)	chr22(−)	*WT1*	*WTX (AMER1)*
No-relapse (*n* = 31)	5	11	7	6	7	6	7	6	5	5	2	2	2
frequency	16.1 %	35.5 %	22.6 %	19.3 %	22.6 %	19.3 %						6.4 %	6.4 %
Relapse (*n* = 19)	3	12	4	5	6	6	3	3	4	2	1	4	1
frequency	15.8 %	*63.2* %^a^	21.0 %	26.3 %	31.6 %	31.6 %						*21.0* %^a^	5.3 %

^a^Significant differences between WTs derived from patients who later relapsed or not

Focal 1q proximal gains were found in 63 % of the WT relapse group and in a significantly lower proportion in the WTs without relapse group (35.3 %). Focal losses encompassing either the *WT1* or *WTX* (*AMER1*) genes (Additional file 2: Figure S1) occurred in 12 and 6 % of WTs, respectively. The frequency of *WT1* deletions was significantly higher in WTs that later relapse than in the group without relapse (21 versus 6.4 %); one tumor from a patient who relapsed was found to carry both *WTX* and *WT1* deletions.

Twelve minimum common regions (MCRs) of chromosomal alterations detected in at least two WTs were identified and are described in Table 2.

**Table 2:** Twelve minimum common regions of chromosomal aberrations detected in Wilms tumours

Genomic coordinates (GRCh37)	Cytoband	Lenght (Mb)	Copy number type
#chr1:23,362,908-26,746,259	1p36.12p36.11	3.38	deletion
#chr1:144,824,185-161,067,947	1q21.1q23.2	16.24	amplicon
#chr2:224,323,183-236,091,182	2q37	11.77	deletion
#chr4:114,506,621-115,974,843	4q26	1.47	deletion
#chr7:39,742,350-49,836,566	7p14.1p12.2	10.10	deletion
#chr7:133548462-158674912	7q33q36.3	25.13	gain
#chr10:125,525,789-135,524,747	10q26.13q26.3	10.00	gain
#chr11:33,713,698-36,511,648	11p13p12	2.80	deletion
#chr11:111,359,499-120,743,656	11q23.1q23.3	9.38	deletion
#chr14:19,373,243-34,950,464	14q11.1q13.1	15.58	gain
#chr14:68,882,507-92,887,911	14q24.1q32.12	24.01	deletion
#chr16:71,201,074-90,294,753	16q22.1q24.3	19.09	deletion

We performed a comparative analysis of differential CNAs looking for the smallest common regions of aberrations that were more frequent in each WT group (tumors derived from patients who later presented relapse or not). In the manual curated analysis, we detected a region of 44.5 Mb at 1q21.1q31.1 (#chr1:144,053,035-188,589,610; GRCh37) that exhibited a higher frequency of gain in the relapse group than in the non-relapse group. This region encompasses the MCR of high-copy number gain at 1q21.1q23.2 (16.2 Mb), which was detected in two of the relapse WTs (*WT1104* and *WT1232*); gains of two of the affected genes, *S100A4* and *NOTCH2* were validated by qPCR in several tumors (Additional file 3: Figure S2). Additionally, we narrowed 14q genomic deletions to a segment at 14q24.1q32.12 common to four WTs: one relapsed tumor (*WT1232*), and three non-relapse cases (*WT095*, *WT246*, and *WT321*).

### Focal chromosomal rearrangements

**Fig. 2:**
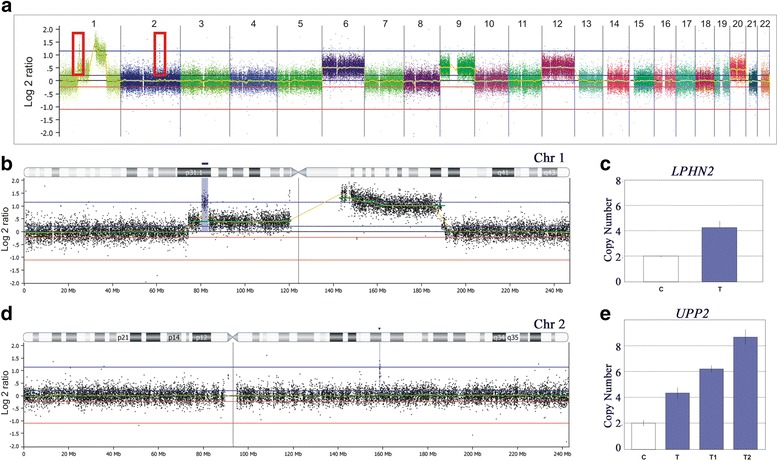
Genomic amplifications detected in one Wilms Tumour. **a** Genomic array-CGH profile of one Wilms tumor (*WT1104*) showing amplifications located in 1p and 2q (*red boxes*). **b** In the chromosome 1 ideogram, the *blue box* marks a 2.4 Mb 1p31.1 amplification containing only one coding gene, *LPHN2*, and underneath is the array-CGH profile of the genomic region. **c** DNA copy number evaluation of the *LPHN2* by qPCR; the *blue bar* represents the tumor sample and the *white bar* the control. Each bar represents the average copy number of 3 replicates, and the *error bars* show the standard deviation (adapted from CopyCaller software, Applied Biosystems). **d** In the chromosome ideogram, the *blue box* marks a 300 kb amplification at 2q24.1, and underneath is the array-CGH profile of the genomic region. **e** DNA copy number evaluation of the *UPP2* by qPCR; the *blue bars* represent three tumor samples and the white bar represents the control. Each bar represents the average copy number of 3 replicates, and the *error bars* show the standard deviation (adapted from CopyCaller software, Applied Biosystems)

Two small regions exhibiting high-copy gains (log_2_ ratio >1.4 indicating >5 copies) were detected in one relapsed WT (*WT1104*), and validated by qPCR: a 2.4 Mb 1p31.1 amplification (#chr1:80,765,303-83,606,627; GRCh37) containing only *LPHN2* (Fig. 2a, b, and c), and a 300 Kb amplification at 2q24.1 (#chr2:158,834,824-159,135,178; GRCh37), encompassing only the *UPP2* and *CCDC148* genes (Fig. 2a, d, and e).

**Fig. 3:**
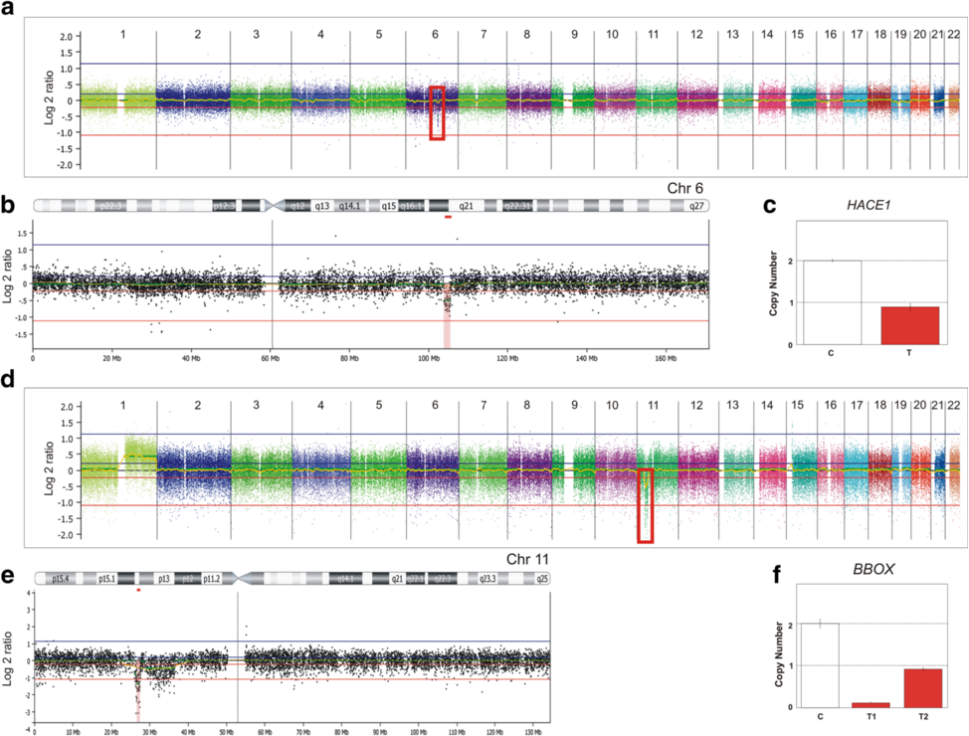
Genomic deletions detected in Wilms tumours. **a** Genomic array-CGH profile of one Wilms tumour (*WT329*) showing the heterozygous loss detected at 6q16.3q21 (*red box*). **b** In the chromosome 6 ideogram, the *red bar* marks a 1.6 Mb microdeletion, and underneath is the array-CGH profile of the region. **c** DNA copy number evaluation of the *HACE1* by qPCR; the *red bar* represents the tumour sample and the *white bar* represents the control. Each bar represents the average copy number of 3 replicates, and the *error bars* show the standard deviation (adapted from CopyCaller software, Applied Biosystems). The results show the presence of a single copy of the *HACE1* sequence in the tumour genome compared to controls. **d** Genomic array-CGH profile of one Wilms tumour (*WT201*) showing the homozygous deletion detected at 11p13p12 (*red box*). **e** In the chromosome 11 ideogram, the *red bar* marks an 825 kb homozygous deletion, and underneath is the array-CGH profile of the region. **f** DNA copy number evaluation of the *BBOX* by qPCR; the *red bars* represent the tumours samples and the *white bar* represents the control. Each bar represents the average copy number of 3 replicates, and the *error bars* show the standard deviation (adapted from CopyCaller software, Applied Biosystems)

Regarding small genomic losses, the 1.6 Mb deletion at 6q16.3q21 (#chr6:103,820,062-105,461,750; GRCh37) encompassed only the genomic sequences of the *HACE1*, *LINC00577*, and *LIN28B* genes (Fig. 3a, b, and c), detected in two non-relapse WTs (*WT329* and *WT1070*), neither carrying *WT1* or *WTX* deletions. Another validated focal CNA detected in one tumor (*WT201*) was the 825 kb homozygous deletion at 11p14.1p14.2 (#chr11:26,688,179-27,513,817; GRCh37), harbouring *BBOX*, among others genes (*SLC5A12*, *FIBIN*, *CCDC34*, *LGR4*) (Fig. 3d, e, and f).

### Gene expression analysis

To evaluate the expression of genes affected by CNAs in these WTs, we selected a set of 90 genes mapped in MCRs, focal rearrangements, and the 1q21.1q23.2 region associated with relapse. There were 35 (39 %) differentially expressed genes between WTs and differentiated kidneys (fold-change ≥|2|; *p* ≤0.05), with only 16 (46 %) exhibiting a concordant pattern of gene expression and type of CNA (see Table 3). Unsupervised hierarchical clustering (Additional file 4: Figure S3) based on the expression pattern of this set of 16 genes discriminated all WT samples from all but one differentiated kidneys (DKs); however, this group of genes was not able to discriminate WT samples with or without later relapse.

**Table 3:** Sixteen differentially expressed genes in the group of Wilms tumours compared to differentiated kidneys which exhibited a concordant pattern of copy number alteration (CNA)

Gene name	Fold change	Type of CNA
*CNKSR1*	−2.30*	1p36.12p36.11 deletion
*MAN1C1*	−7.37**	1p36.12p36.11 deletion
*PAQR7*	−10.71**	1p36.12p36.11 deletion
*CHD1L*	3.18**	1q21.1q23.2 gain
*CRABP2*	31.59**	1q21.1q23.2 gain
*GJA8*	25.48*	1q21.1q23.2 gain
*MEX3A*	10.28**	1q21.1q23.2 gain
*MLLT11*	14.04**	1q21.1q23.2 gain
*DNMT3A*	2.42**	2p25.3p11.2 gain
*INPP5D*	−2.56*	2q37 deletion
*AHR*	−9.62**	7p14.1p12.2 deletion
*SOSTDC1*	−21.39**	7p14.1p12.2 deletion
*TWIST1*	−3.26*	7p14.1p12.2 deletion
*BBOX1*	−39.84*	11p13p12 homozygous loss in one WT
*FIBIN*	−8.10*	11p13p12 homozygous loss in one WT
*PLCG2*	−8.96*	16q22.1q24.3 deletion

*p < 0.05, **p < 0.01

Eleven genes located at genomic deletions were found to be down-regulated in WTs when compared with DKs, nine of them mapped in MCRs: *MAN1C1*, *CNKSR1*, and *PAQR7* (1p36); *INPP5D* and *ECEL1* (2q37); *SOSTDC1*, *TWIST1* and *AHR* (7p14.1p12.2); and *PLCG2* (16q22.1q24.3). The remaining two down-regulated genes, *BBOX* and *FIBIN*, were mapped in a homozygous 11p13 deletion detected in a single tumor.

Regarding those genes located at copy number gains, five of them, mapped at the 1q21.1q23.2 gain (more frequently detected in relapsed samples), were found to be over-expressed in WTs compared with DKs: *CHD1L*, *CRABP2*, *GJA8*, *MEX3A*, and *MLLT11*.

## Discussion and conclusions

In this study, WTs exhibited a relatively small number of CNAs indicating low chromosomal instability, in accordance with previous reports of favourable histology WTs. Primary WTs that later relapse are supposedly more aggressive as they are resistant to chemotherapeutic treatments. The fact that these tumors displayed more chromosome alterations and higher gains is in part supported by findings of a high level CNAs found in the more aggressive diffuse anaplastic WT subtype [32]. Most of the chromosome alterations from this study has already been described by previous studies, such as 1q gain, and 11q and 16q losses [23, 27, 30] as well as alterations reported in low frequency, including the 2q37 deletion [16], and the 6q21 deletion [15]. However, a few CNAs detected in frequencies >15 % were reported here for the first time in WT, including 7q and 20q gains, and 7p loss.

In this study, the blastemal component of WT was micro-dissected before DNA extraction. Eventually, this procedure could minimize findings related to tumor heterogeneity that have been described in pediatric tumors [33], at least those related to cell differentiation. Unfortunately, the array-CGH platform used in this study does not allow allelic identification, impeding a careful assessment of the presence of intratumor heterogeneity at low levels.

The association between chromosomal alterations and cancer recurrence in patients with WT has been suggested by some publications, all of which share similar findings [25, 27, 34, 35]. In our samples, the 1q gain was found in 63.2 % of the tumors that later relapse in comparison with 35.5 % of the non-relapsed WTs. This higher frequency of 1q gain, compared to previous studies, can be explained by the fact that in our tumor series only the blastemal component were assessed. A specific genomic segment at 1q21.1q31.3 has been associated with WT relapse [28, 36], and in the present work this region was narrowed to a 16.2 Mb segment at 1q21.1q23.2. Five genes mapped within this 1q21.1q23.2 segment were found to be up-regulated in WTs compared with DKs (*CHD1L*, *CRABP2*, *GJA8*, *MEX3A* and *MLLT11*) but not between relapse and no-relapse WT. *CRABP2* higher expression also had a weak association with high stage WTs [37]. *MLLT11* has a role with leukemogenesis [38, 39], and *CHD1L* is over-expressed in hepatocellular carcinomas [40].

Combined LOH of 16q and 1p is a known marker of poor prognosis; indeed, we found a higher frequency of 16q deletion (31.6 %) in relapsed cases when compared with non-relapsed cases (19.3 %), and narrowed to a 19 Mb segment at 16q22.1q24.3. We also found deletions affecting *WT1* in 12 % of all cases detected with a significantly higher frequency in the relapse group. Although these genomic features could be useful as prognostic indicators for blastemal predominance, they were very infrequent events, therefore adding little to the ability to distinguish patients with different outcomes.

Losses of 14q have been shown to have a borderline association with tumor stages III and IV [27] suggesting that genes located in this chromosome region have the potential to be involved in tumor progression. WTs here studied are mostly stages III and IV tumors, and the detection of 14q deletions in four samples allowed to narrow this genomic deletion to a segment at 14q24.1q32.12 (one relapsed and three non-relapse cases).

Other MCRs of new genomic deletions highlighted few genes with a concordant profile with gene expression, particularly *CNKSR1*, *MAN1C1* and *PAQR7* at 1p36, and *TWIST1* and *SOSTDC1* at 7p14.1p12.2, the later already  suggested as tumor suppressor in WT [41]. The analysis of other small alterations of high amplitude in copy number change disclosed new candidate genes potentially associated with WT such as *LPHN2*, *UPP2*, *BBOX* and *FIBIN*. Only *BBOX* and *FIBIN*, identified in a homozygous deletion at 11p13, exhibited a concordant down-regulated pattern of gene expression when considering the entire WT group; therefore, both genes appear as candidate tumor suppressor genes for WT but need further studies to confirm their role.

We are aware that this study has limitations. The set of genes which expression were compared to differentiated kidneys, not an ideal control, came from alterations found in only one or few tumors, thus explaining the observed low level of agreement between CNAs and expression pattern. Additionally, we do not have tested other patient’s tissues to exclude the possibility that part of the detected CNAs were germline alterations. However, all CNAs were checked in the Database of Genomic Variants and none of them was found to be common changes.

In summary, most of the detected CNAs in this study were described by previous works. However, the present work identified that genomic amplifications and higher number of genomic losses occur in tumors that later relapsed. Additionally, these tumors exhibited an increased frequency of a gain of a 16.2 Mb segment at 1q21.1q23.2, and losses at 11p, 11q distal, and 16q, together with *WT1* deletions. Conversely, aneuploidies of chromosomes 13 and 19 (whole-chromosome gains) were exclusively detected in WTs without relapse, suggesting that these are good prognosis markers.

The CNAs affected the expression of few genes (over-expression of *CHD1L*, *CRABP2*, *GJA8*, *MEX3A* and *MLLT11* and down-regulation of *CNKSR1*, *MAN1C1*, *PAQR7*, *TWIST1*, *SOSTDC1*, *BBOX* and *FIBIN*) that could have an oncogene or tumor suppressor role in WT. We stress that although the studied cohort of WTs is small, most of the genomic regions here identified have been described in WT by others, reinforcing that they should be investigated in depth to disclose the possible roles of the affected genes in Wilms tumorigenesis.

While isolated genes can account for selection of specific chromosome imbalances (drivers), another alternative theory, applying an evolutionary perspective, hypothesizes that the different karyotypes with specific combinations of chromosome alterations could result in slightly different tumor  subtypes. The high rate of variation within a tumor generates tumor sub-clones with different phenotypic progression, as exemplified by the acquisition of resistance to chemotherapy or the metastatic growth [42, 43]. For instance, in the case of WT, this process could also be reflected by the heterogeneous histology found within each tumor. Some preliminary data reported intra-tumor heterogeneity in 70 % of the WT cases albeit they may also share some common copy number changes [44]. Most of the alterations we found are shared by WTs from other studies, suggesting this is a relatively common route for Wilms tumorigenesis.

## Methods

### Material

Samples of sporadic primary WTs were obtained from 50 patients enrolled in the National Wilms tumor Study 5 (NWTS-5, Children Oncology Group). The group is enriched for WTs stages III and IV, which characteristics have been described [45]. Tumors analysed were not subjected to neoadjuvant chemotherapy; 31 of these patients exhibited no relapse after a minimum of 3 years of follow-up. All samples were obtained with informed consent. This work was conducted in accordance with the principles of the Declaration of Helsinki and was approved by the A. C. Camargo Cancer Center ethics committee under number CEP 764/06.

### Comparative genome hybridization based on microarrays (array-CGH)

We performed comparative genomic hybridization based on microarrays in a commercial whole-genome 180 K platform containing 180,000 oligonucleotide probes (Agilent Technologies; design 22060). Reference DNA was a commercially available human pool of samples from healthy donors (Promega). Briefly, samples were labelled with Cy3- or Cy5-deoxycytidine triphosphates by random priming, and purification, hybridization and washing were performed as recommended by the manufacturer. Scanned images of the arrays were processed using Feature Extraction 10.7.3.1 software (Agilent Technologies).

Array-CGH analysis was performed using Nexus Copy Number software 7.0 (Biodiscovery) with the FASST2 segmentation algorithm, according to the following settings: minimum of 5 consecutive probes (effective resolution of ~70 Kb for CNA calling), significance threshold set at 10^−8^, and threshold log_2_ Cy3/Cy5 of 0.3 and 1.4 for gain or high copy gain (indicating >5 copies of the genomic sequence, and hereafter named amplification), respectively, and −0.3 and −1.1 for loss and homozygous loss, respectively. All copy number variants reported in the Database of Genomic Variants (DGV; http://dgv.tcag.ca/dgv/app/home) were excluded, as well data from sex chromosomes; the X-linked gene *AMER1* (*WTX*) was analysed separately. The minimum common regions (MCRs) of recurrent CNAs were obtained by implementing the global frequency statistical approach of the STAC method (Significance Testing for Aberrant Copy Number [46]). Data were evaluated iregarding the total number of CNAs and the numbers of gains, losses, amplifications, and homozygous losses. Genes affected by copy number changes were annotated using the Genome browser UCSC (http://genome.ucsc.edu/). Statistical analyses were performed using the software GraphPad PRISM 5.

### DNA copy number validation by real-time quantitative PCR (qPCR)

To validate 9 focal CNAs (<5 Mb), we performed qPCR using 9 TaqMan probes (see Additional file 5: Table S3) on a 7500 Fast Real-time quantitative PCR System (Applied Biosystems). Copy number determinations were performed for selected targets using TaqMan Gene Copy Number Assays (Applied Biosystems). The assays contained a FAM-labelled TaqMan probe for the target gene and a VIC-labelled TaqMan probe for the reference gene (RNaseP). The reference sample or calibrator was a commercially available human genomic DNA (Promega). The results were analysed using CopyCaller 1.0 software (Applied Biosystems). The relative number of DNA copies for each probe was determined by the DDCt ((FAM Ct–VIC Ct) sample–(FAM Ct–VIC Ct) calibrator) method, which assumes that the calibrator DNA has two copies of the reference gene.

### Gene expression analysis by reverse transcription quantitative real-time PCR (RT-qPCR)

We selected 90 genes for gene expression evaluation by RT-qPCR (Additional file 6: Table S4) that were affected by recurrent copy number changes in our cohort of WTs. Total RNA samples were enzymatically converted into first-strand cDNA using an RT^2^ First Strand cDNA Kit (Qiagen). We evaluated 36 WT blastemal enriched samples and six differentiated kidneys (DK) used as controls. These control samples constituted the cortex of differentiated kidneys from nephrectomies of WT patient’s macrodissected after evaluation of hematoxilin-eosin sections. We used a SYBR-green based customized array RT^2^ qPCR Primer Customized Assay (Qiagen Technologies) following the manufacturer’s protocol. RT-qPCR was performed in an ABI Prism 7900HT Fast Real-time Sequence Detection System (Life Technologies, Foster City, CA). *ACTB*, *GAPDH*, and *HPRT1* were tested as reference genes, and the two most stable genes (as determined by geNorm [47]), namely, *ACTB* and *GAPDH*, were used for normalization in the expression analysis. The array data were analysed by SDS and RQ manager (Life Technologies), and gene expression normalization was calculated using the 2ΔCq method. Genes were considered differentially expressed between groups (WTs and DKs) if the fold change was ≥|2| with *p*-value ≤0.05 (student *t*-test).

## Additional files

Additional file 1: Table S1. Full CNA data identified by array-CGH in the 50 Wilms tumor samples. CNA calling was performed using the software Nexus Copy Number 7.0 (Biodiscovery). **Table S2.** Array-CGH data summary of Wilms tumor samples: total number of copy number alteration, number of each type of copy number event (gain, loss, high-copy gain, and homozygous loss), and statistical analysis. (XLS 872 kb) Additional file 2: Figure S1. DNA copy number evaluation showing focal and homozygous losses of the WT1 (A) and WTX (B) genes by qPCR; the red bars represent tumour samples and the white bar represents the control. Each bar represents the average copy number of 3 replicates, and the error bars show the standard deviation (adapted from CopyCaller software, Applied Biosystems). (TIF 226 kb) Additional file 3: Figure S2. DNA copy number evaluation showing amplification of S100A4 (A) and NOTCH2 (B) genes at 1q21.1-q23.2 in several tumours by qPCR; the blue bars represent tumour samples and the white bar represents the control. Each bar represents the average copy number of 3 replicates, and the error bars show the standard deviation (adapted from CopyCaller software, Applied Biosystems). (TIF 349 kb) Additional file 4: Figure S3. Unsupervised hierarchical clustering using Pearson’s correlation, and complete linkage of 36 Wilms tumour (WTs) and 6 differentiated kidney (DKs) samples based on 16 differently expressed genes (values were log2-transformed). Only genes with expression detected in more than 80 % of the samples were considered. Bootstrap resampling was performed to assess cluster reliability, and the results are represented by the coloured lines of the dendrogram (black line indicates 90–100 % reliability). Differentiated kidney samples are marked in blue, and Wilms Tumour samples are coloured pink (light pink are non-relapse samples, and dark pink are relapse samples). Columns and rows represent samples and genes, respectively; red, upregulated, and green, down-regulated genes. (TIF 425 kb) Additional file 5: Table S3. List of genes selected for copy number validation of 9 focal rearrangements using real-time quantitative PCR (qPCR) with TaqMan Gene Copy Number assays. (DOC 34 kb) Additional file 6: Table S4. Description of 90 genes that were selected for gene expression analysis in the group of Wilms tumors using a SYBR-green based customized array RT^2^ qPCR Primer Customized Assay (Qiagen Technologies). Description of the genes selected as controls for gene expression analysis in the group of Wilms tumors using a the SYBR-green based customized array RT^2^ qPCR Primer Customized Assay (Qiagen Technologies). (XLS 56 kb)
