# Supplementary figures and images for: Genomic imbalances pinpoint potential oncogenes and tumor suppressors in Wilms tumors
Source: Mol Cytogenet. 2016 Feb 24;9:20. doi: 10.1186/s13039-016-0227-y (PMC4765068; doi:10.1186/s13039-016-0227-y)

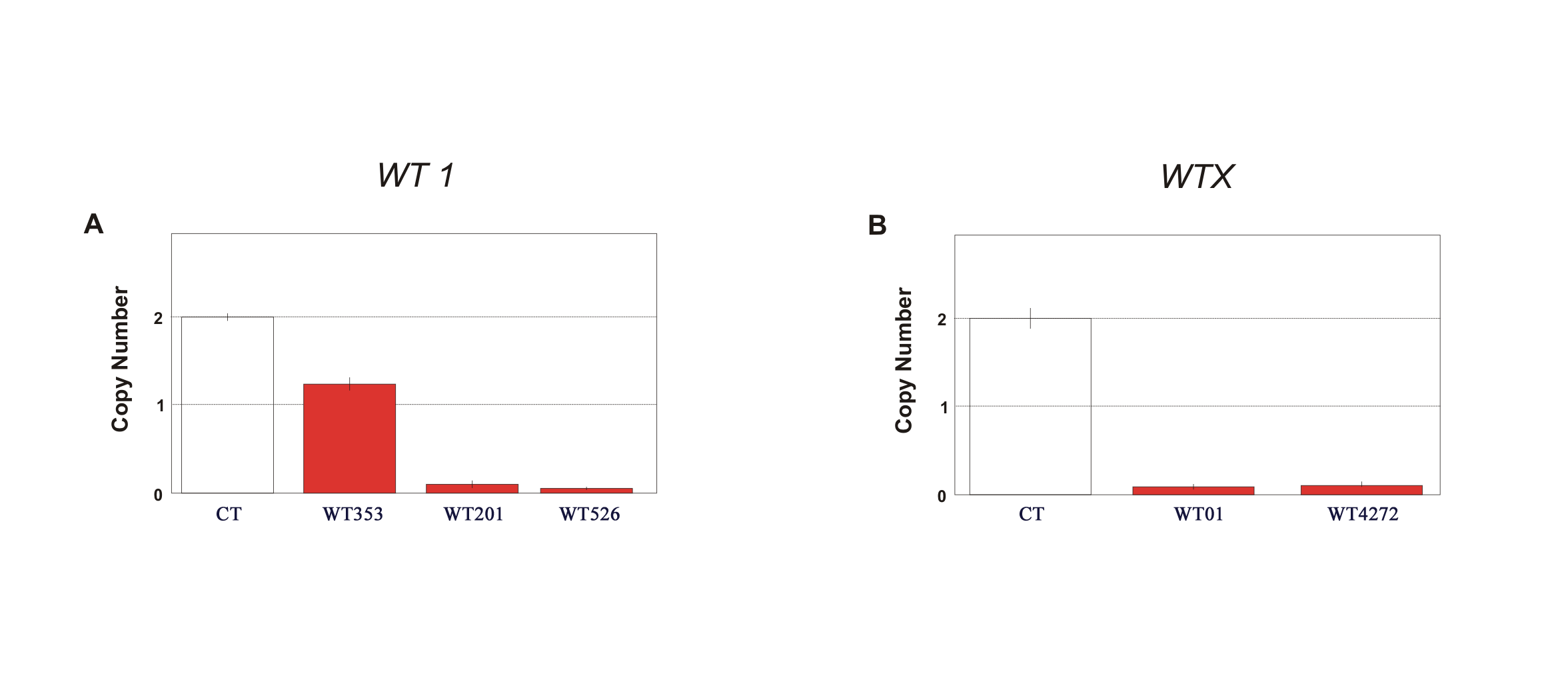

Supplement: Additional file 2: Figure S1. — DNA copy number evaluation showing focal and homozygous losses of the WT1 (A) and WTX (B) genes by qPCR; the red bars represent tumour samples and the white bar represents the control. Each bar represents the average copy number of 3 replicates, and the error bars show the standard deviation (adapted from CopyCaller software, Applied Biosystems). (TIF 226 kb) [file 13039_2016_227_MOESM2_ESM.tif]

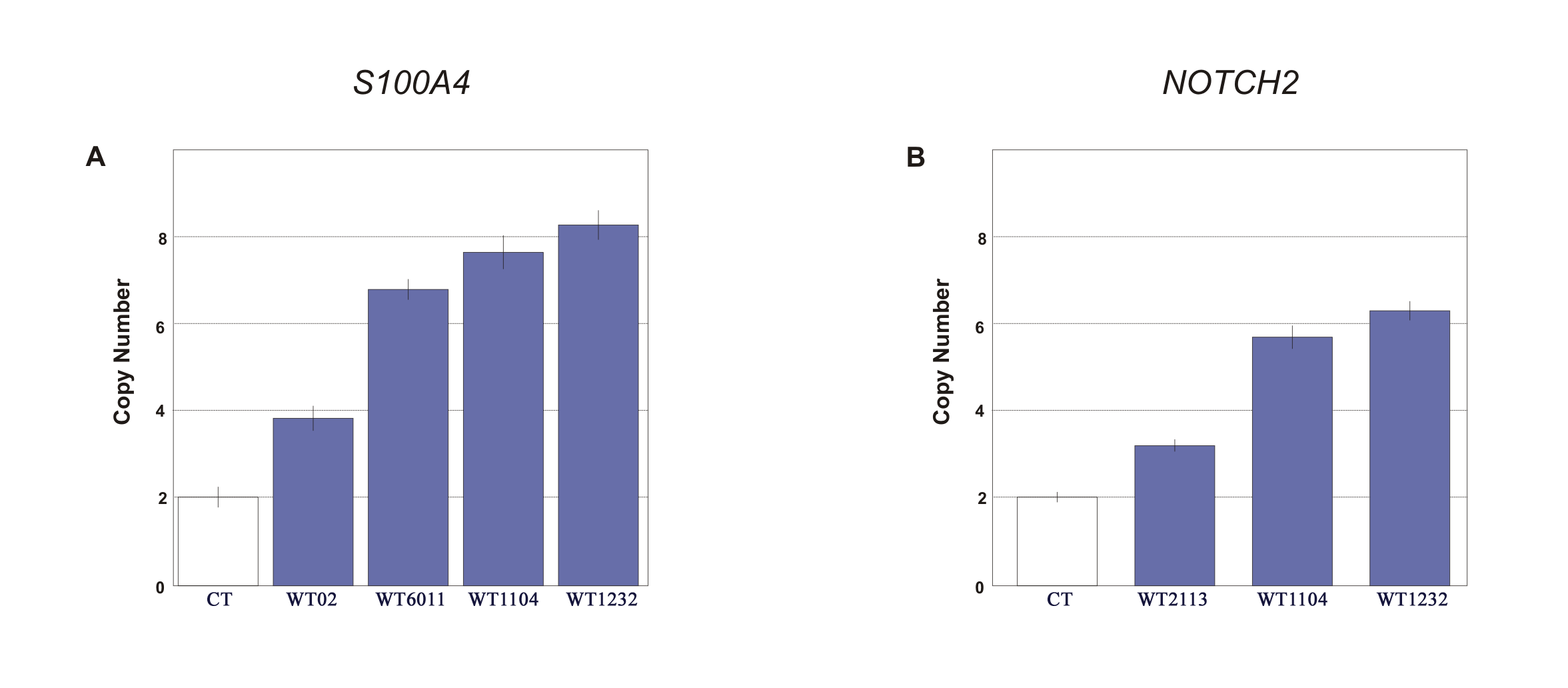

Supplement: Additional file 3: Figure S2. — DNA copy number evaluation showing amplification of S100A4 (A) and NOTCH2 (B) genes at 1q21.1-q23.2 in several tumours by qPCR; the blue bars represent tumour samples and the white bar represents the control. Each bar represents the average copy number of 3 replicates, and the error bars show the standard deviation (adapted from CopyCaller software, Applied Biosystems). (TIF 349 kb) [file 13039_2016_227_MOESM3_ESM.tif]

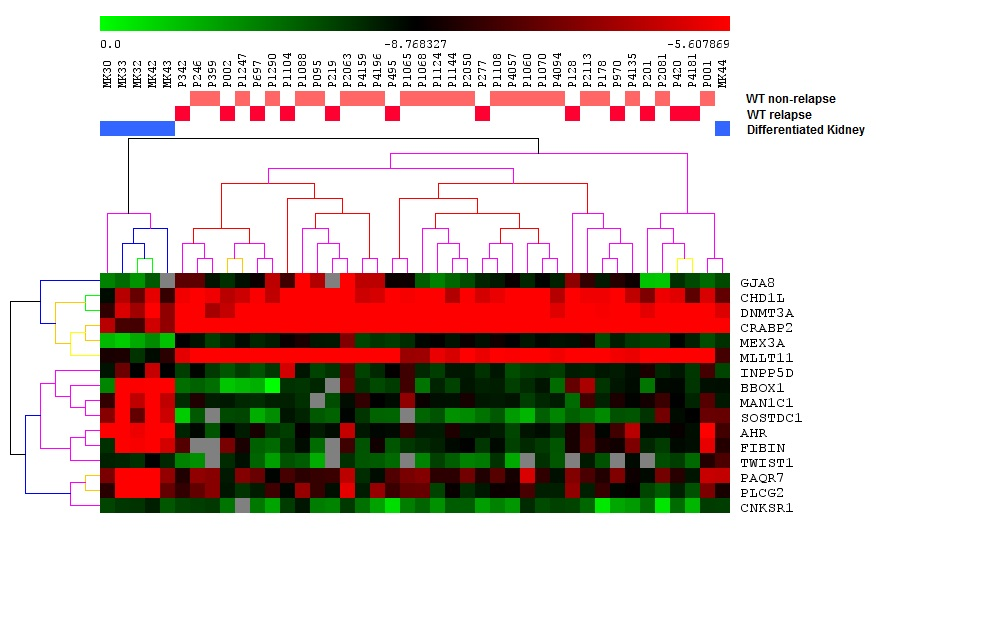

Supplement: Additional file 4: Figure S3. — Unsupervised hierarchical clustering using Pearson’s correlation, and complete linkage of 36 Wilms tumour (WTs) and 6 differentiated kidney (DKs) samples based on 16 differently expressed genes (values were log2-transformed). Only genes with expression detected in more than 80 % of the samples were considered. Bootstrap resampling was performed to assess cluster reliability, and the results are represented by the coloured lines of the dendrogram (black line indicates 90–100 % reliability). Differentiated kidney samples are marked in blue, and Wilms Tumour samples are coloured pink (light pink are non-relapse samples, and dark pink are relapse samples). Columns and rows represent samples and genes, respectively; red, upregulated, and green, down-regulated genes. (TIF 425 kb) [file 13039_2016_227_MOESM4_ESM.tif]
