# Supplementary material for: Genomic imbalances pinpoint potential oncogenes and tumor suppressors in Wilms tumors
Source: Mol Cytogenet. 2016 Feb 24;9:20. doi: 10.1186/s13039-016-0227-y (PMC4765068; doi:10.1186/s13039-016-0227-y)
Supplement: Additional file 5: Table S3. — List of genes selected for copy number validation of 9 focal rearrangements using real-time quantitative PCR (qPCR) with TaqMan Gene Copy Number assays. (DOC 34 kb) [file 13039_2016_227_MOESM5_ESM.doc]

**Supplementary Table S3.** List of genes selected for copy number validation of 9 focal rearrangements using real-time quantitative PCR (qPCR) with TaqMan Gene Copy Number assays.

| **Gene (official name)** | **Cytoband** | **Exon** | **Probe genomic mapping** (GRCh37) | **Array-CGH data** |
| --- | --- | --- | --- | --- |
| *NOTCH2* | 1p12 | 5 | chr1:120529672 | Gain |
| *LPHN2* | 1p31.11 | 3 | chr1:158958642 | Gain |
| *LIN28A* | 1p36.11 | 1 | chr1:26737300 | Loss |
| *S100A4* | 1q21.3 | 3 | chr1:153517211 | Gain |
| *UPP2* | 2q24.1 | 3 | chr2:158958642 | Gain |
| *HACE1* | 6q21 | 12 | chr6:105232860 | Loss |
| *WT1* | 11p13 | 6 | chr11:32421634 | Loss |
| *BBOX* | 11p14.2 | 7 | chr11:27141305 | Loss |
| *WTX (AMER1)* | Xq11.1 | 2 | chrX:63404997 | Loss |
